# Clinical Characteristics and Timing of Accessory Pathway Reconduction During Catheter Ablation of Accessory Pathways

**DOI:** 10.1002/joa3.70469

**Published:** 2026-09-18

**Authors:** Wataru Sasaki, Hitoshi Mori, Daisuke Kawano, Kei Matsumoto, Naomichi Tanaka, Tsukasa Naganuma, Masataka Narita, Kazuhisa Matsumoto, Yoshifumi Ikeda, Takahide Arai, Shintaro Nakano, Ritsushi Kato

**Affiliations:** ^1^ Department of Cardiology Saitama Medical University International Medical Center Hidaka Saitama Japan

**Keywords:** accessory pathway, catheter ablation, recurrence, supraventricular tachycardia, waiting period, WPW syndrome

## Abstract

**Background:**

Catheter ablation of accessory pathways has high acute success rates; however, accessory pathway (AP) recurrence remains a clinically relevant issue. Although intra‐procedural AP reconduction is frequently encountered during ablation procedures, its timing, predictors, and clinical significance remain insufficiently characterized.

**Objectives:**

This study aimed to investigate the timing, electrophysiologic characteristics, and clinical implications of intra‐procedural AP reconduction during catheter ablation of manifest and concealed accessory pathways.

**Methods:**

We retrospectively analyzed 128 patients with manifest or concealed AP who underwent catheter ablation at our institution between May 28, 2015, and February 2025. Details of cases in which AP reconduction was observed during the 30 min waiting period after successful ablation, including clinical and procedural characteristics, were compared with those of patients without reconduction.

**Results:**

Intra‐procedural AP reconduction occurred in 23 patients (18.0%). The mean time to reconduction was 16.7 ± 7.0 min, with a maximum interval of 27 min. Compared with patients without reconduction, those with reconduction had a significantly longer time to pathway interruption, more frequent broad or oblique APs, and more complex procedural approaches requiring multiple anatomical access routes. Intra‐procedural reconduction was also associated with subsequent late recurrence. Fifteen of 23 events (65.2%) occurred ≥ 15 min after AP elimination.

**Conclusions:**

Intra‐procedural AP reconduction during catheter ablation of accessory pathways is not uncommon and appears to be associated with specific electrophysiologic and procedural characteristics as well as late recurrence. These findings may facilitate identification of patients at higher risk of reconduction and help guide intra‐procedural assessment during catheter ablation.

## Graphical abstract

Intra‐procedural accessory pathway reconduction occurred in 18% of patients, with 65.2% of events detected ≥ 15 min after initial pathway elimination. Reconduction was associated with a higher rate of late recurrence, supporting careful assessment for conduction recovery during the post‐ablation waiting period.

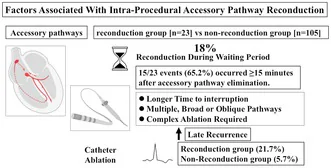

**Abbreviations**
AP: accessory pathway
AVRT: atrioventricular reentrant tachycardia
CEA: continuous electrical activity
CS: coronary sinus
ECG: electrocardiogram
IECG: intracardiac electrogram
MVA: mitral valve annulus
RF: radiofrequency
RV: right ventricle

## 1 Introduction

Catheter ablation is an established and highly effective treatment for patients with accessory pathways, including both manifest and concealed pathways, achieving high acute success rates and favorable long‐term outcomes. Nevertheless, recurrence of accessory pathway (AP) conduction remains a clinically relevant issue, occurring even after apparently successful ablation. Previous studies have primarily focused on long‐term recurrence rates and predictors of procedural success [1], whereas mechanisms contributing to recurrence during the ablation procedure itself remain incompletely understood.

In clinical electrophysiology practice, intra‐procedural AP reconduction is not uncommon after apparent elimination of pathway conduction and frequently prompts additional radiofrequency applications. However, the timing, electrophysiologic characteristics, and procedural predictors of intra‐procedural reconduction have not been systematically evaluated. As a result, there is limited evidence to guide procedural decision‐making, including the optimal duration of post‐ablation waiting periods and identification of patients at higher risk of reconduction.

Accordingly, we sought to investigate the incidence, timing, electrophysiologic characteristics, and clinical implications of intra‐procedural AP reconduction during catheter ablation of manifest and concealed accessory pathways. We additionally aimed to identify procedural predictors of reconduction and evaluate its association with subsequent late recurrence.

## 2 Methods

### 2.1 Study Patients

We retrospectively analyzed 128 patients with manifest or concealed accessory pathways who underwent catheter ablation at our institution between July 2, 2015, and February 26, 2025. Both index and repeat ablation procedures were eligible for inclusion. Patients in whom AP elimination was not achieved during the procedure, those younger than 15 years of age, and patients with congenital heart disease who had undergone prior open‐heart surgery were excluded from the analysis. Identification of AP localization from the 12‐lead ECG was guided by the electrocardiographic algorithm developed and validated by Arruda et al. [2].

After successful ablation of the APs, all patients underwent a 30 min waiting period, which was defined as the interval from the last ablation application to the end of the procedure. If AP reconduction was observed during this period, additional ablation was performed, followed by a second 30 min waiting period to reassess for reconduction. Patients who demonstrated AP reconduction during the waiting period were classified as the reconduction group, whereas those without reconduction were classified as the non‐reconduction group. In cases where reconduction occurred, the time to reconduction, together with clinical and procedural characteristics, was compared to patients without reconduction. Reconduction of the APs in the reconduction group was investigated, and clinical data were compared between patients with and without reconduction after the procedure.

Late recurrence was defined as recurrence of AP conduction or AP–mediated supraventricular tachycardia documented by a 12‐lead electrocardiogram or Holter electrocardiographic monitoring during outpatient follow‐up.

This study was performed in accordance with the provisions of the Declaration of Helsinki and local regulations. The research protocol was approved by the hospital's institutional review board (2025–061). Informed consent was obtained using an institutional opt‐out procedure approved by the Institutional Review Board. Information regarding the study was made publicly available on the institutional website, providing participants with the opportunity to decline participation.

### 2.2 Ablation Procedure

All procedures were performed under intravenous anesthesia using continuous infusions of propofol and dexmedetomidine for sedation, with supplemental analgesia provided by intravenous pentazocine as needed. Electrode catheters were inserted via the femoral vein and positioned in the coronary sinus, high right atrium, right ventricular apex, and His bundle region. A comprehensive electrophysiological study was conducted to confirm the presence of an AP and to induce AVRT for a definitive diagnosis [3]. The AP location was also identified using high‐density mapping [4, 5]. Catheter ablation was then performed based on this diagnosis. Radiofrequency (RF) energy delivery was carried out during AVRT, sinus rhythm, or right ventricular (RV) pacing, depending on whether the AP was manifest or concealed, and at the discretion of the operator. RF ablation was the primary energy source, with irrigated‐tip ablation catheters used in the majority of cases. Cryoablation was selected at the operator's discretion in cases where ablation near the atrioventricular node posed a higher risk of atrioventricular block (AVB). Following successful elimination of the AP, continuous intravenous isoproterenol (1–5 μg/min) and programmed stimulation via electrode catheters were used to assess for recurrence. The procedural endpoint was defined as the absence of AP reconduction for 30 min after the final application. The total number of applications was defined as the number of ablation applications delivered before the first detection of accessory pathway reconduction. In patients without reconduction, all ablation applications delivered during the procedure were included.

Patient characteristics, anatomical location of the AP, manifest or concealed nature of the pathway, and procedural parameters were compared between the two groups to identify factors associated with intra‐procedural reconduction. We performed subgroup analyses according to the location of AP (left AP, right AP, and septal AP). Within each type, patients were stratified into reconduction and non‐reconduction groups, and comparisons were made with respect to (1) the location of the accessory pathway and (2) procedural characteristics.

The time to interruption was defined as follows: for antegrade AP ablation, it was the interval from the onset of the RF application to the beginning of the QRS complex in which the Δ wave disappeared on the surface 12‐lead ECG (Figure S1A). For retrograde AP ablation, it was defined as the interval from the onset of the radiofrequency application to the atrial electrogram immediately following AP interruption (Figure S1B). We defined “time to reconduction of the AP” as the interval between the initiation of the waiting period and the initial documentation of AP reconduction. The pattern of reconduction was classified as antegrade‐only, retrograde‐only, or bidirectional according to the direction of recovered AP conduction documented during the waiting period. A complex ablation approach was defined as cases requiring an application from both atrial and ventricular aspects (e.g., supra‐ and sub‐valvular approaches) or from within the coronary sinus (CS).

Based on the electrophysiologic characteristics of successful ablation sites described by Grimm et al. continuous electrical activity (CEA) was used as a key local electrogram marker of AP engagement [6]. In cases of manifest AP in which ablation was performed during sinus rhythm or atrial pacing, the timing of local pathway activation was quantified by measuring the interval from the onset of the surface delta wave to the initiation of CEA (Figure S2A). In cases with retrograde accessory pathway conduction in which ablation was performed during ventricular pacing, the interval from the earliest atrial activation recorded along the atrioventricular annulus to the onset of CEA was obtained (Figure S2B). This interval was defined as the interval to the onset of CEA.

### 2.3 Definition of Broad Accessory Pathways

**FIGURE 1:**
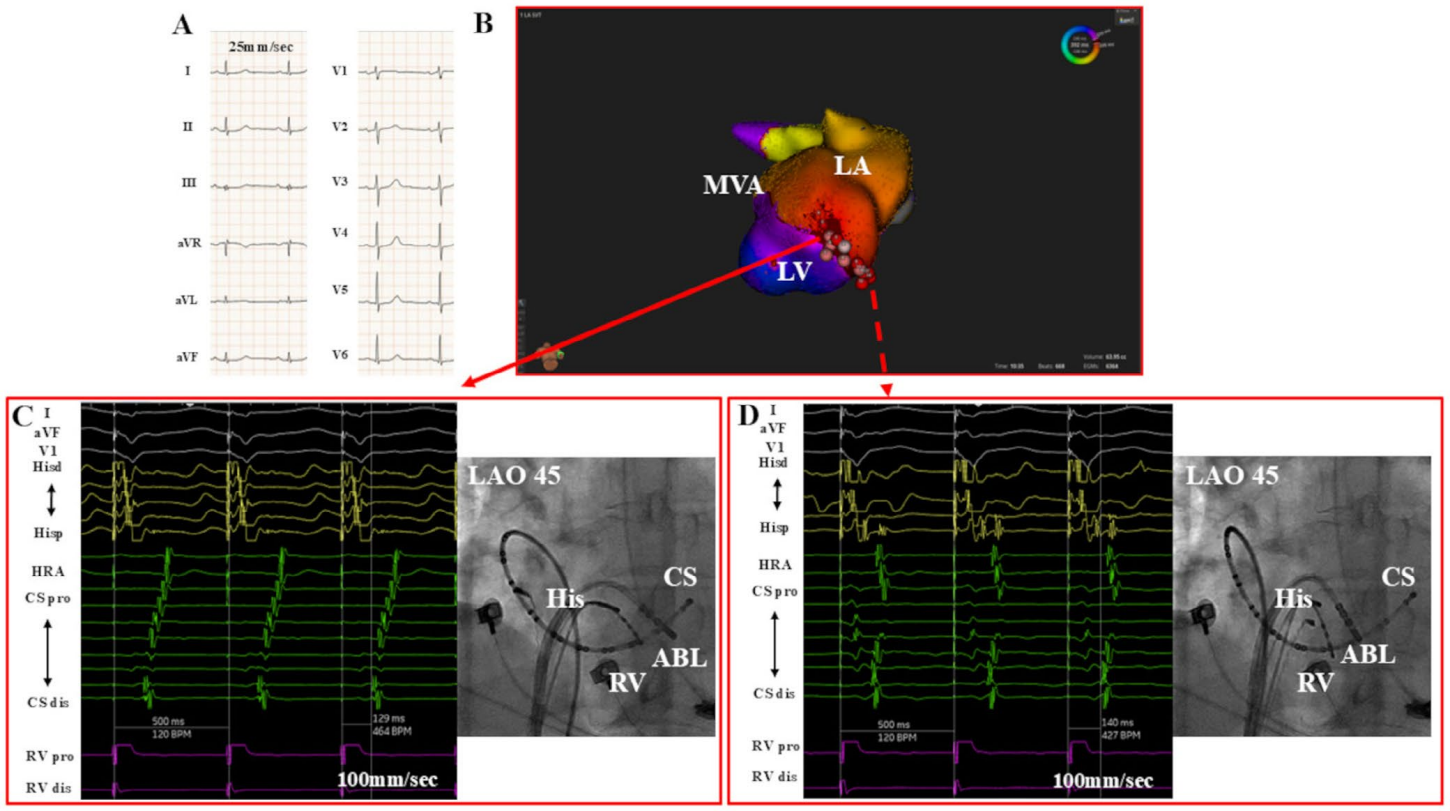
Representative case of a broad accessory pathway. (A) Baseline 12‐lead ECG exhibited no delta waves, consistent with a concealed AP. (B) During RV apex pacing, the earliest atrial activation was broadly observed between the 4 and 5 o'clock positions on the MVA. (C) Left panel shows intracardiac electrograms (IECG) during baseline RV pacing at a cycle length of 500 msec. The earliest atrial activation was observed at CS 2–3, corresponding to the 4–5 o'clock position on the MVA. The stimulus‐to‐VA interval was 129 msec, consistent with a concealed AP. Right panel shows a fluoroscopic image of the ablation catheter positioned at the initial ablation site, indicated by the red arrow. (D) Left panel shows an IECG recorded after multiple RF applications during RV pacing at a cycle length of 500 msec. The earliest atrial activation was still observed at CS 2–3, similar to pre‐ablation findings; however, the stimulus‐to‐VA interval was prolonged to 140 msec compared to baseline. Right panel shows a fluoroscopic image of the ablation catheter positioned at the final ablation site, indicated by the red dotted arrow. CS, coronary sinus; dis, distal; His, his bundle region; HRA, high right atrium; IECG, intra cardiac electrogram; LA, left atrium; LAO 45, left anterior oblique 45° view; LV, left Ventricle; MVA, mitral valve annulus; pro, proximal; RAO 45, right anterior RV, right ventricle; VA, ventriculoatrial.

A broad pathway was defined as a pathway exhibiting prolongation of the local atrioventricular (AV) or ventriculoatrial (VA) interval without complete interruption of the AP, or manifesting changes in the delta wave morphology during the radiofrequency energy delivery [7]. Figure 1 shows a representative case of a concealed broad AP (Figure 1A). During baseline right ventricular (RV) apex pacing, the earliest atrial activation was observed at the 4 o'clock position on the MVA (Figure 1B,C). Dual‐chamber mapping during orthodromic AVRT revealed a broad area of earliest atrial activation spanning from the 4 to 5 o'clock positions on the MVA (Figure 1B, Movie S1). RF energy was initially delivered at the 4 o'clock position during tachycardia (Figure 1C). Although the tachycardia terminated more than 10 s after ablation, the AP was not successfully interrupted. Repeated episodes of interruption and reconduction were observed. Ultimately, ablation across a broader area up to the 5 o'clock position resulted in successful pathway elimination (Figure 1D). Following the successful ablation, RV apex pacing still demonstrated the earliest atrial activation at the 4 o'clock MVA position; however, the ventriculoatrial (VA) interval was approximately 10 msec longer, accompanied by a change in the atrial activation sequence. An additional RF application finally eliminated the VA conduction at the 4 o'clock MVA position. These findings suggested the presence of a broad accessory pathway [7].

### 2.4 Definition of Multiple Accessory Pathways

**FIGURE 2:**
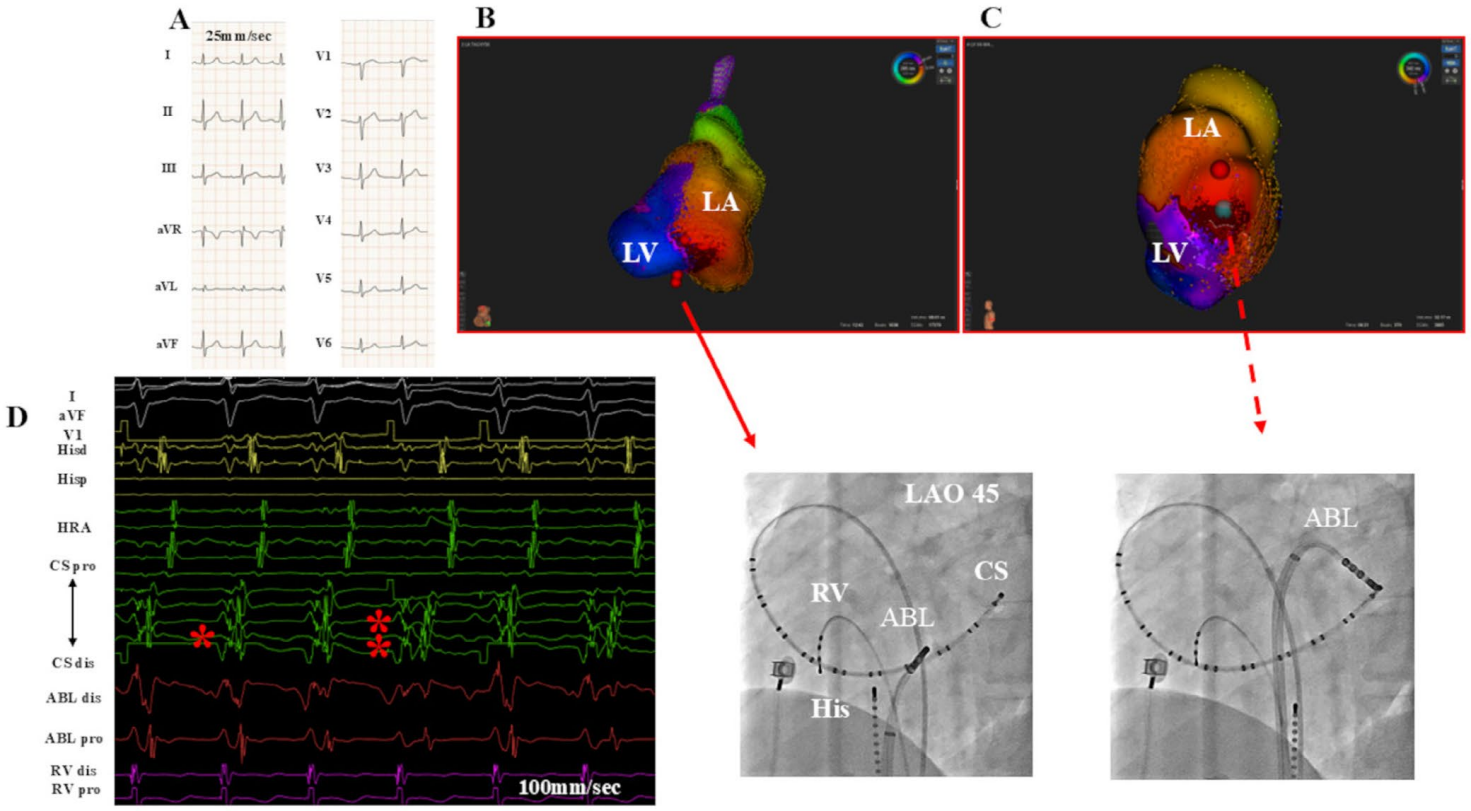
Representative case of multiple accessory pathways. (A) The baseline 12‐lead ECG showed no delta waves, consistent with a concealed AP. (B) A dual‐chamber activation map obtained during AVRT. The red arrow indicates the successful ablation site at the 7 o'clock position on the MVA, along with the corresponding fluoroscopic image showing the catheter position at the time of AP interruption. (C) A dual‐chamber map obtained during RV pacing, demonstrating a second AP at the 3 o'clock position on the MVA. The red dotted arrow marks the successful ablation site for this AP, along with the fluoroscopic image showing the catheter position at the time of pathway elimination. (D) IECG during AVRT, in which the earliest atrial activation was observed at the 7 o'clock position on the MVA. Although RF energy was delivered to this site, the tachycardia did not terminate; however, the site of earliest atrial activation shifted from MVA 7 o'clock (*) to MVA 3 o'clock (⁑). Abbreviations are as in Figure 1.

A multiple pathway was defined as the presence of differing patterns of antegrade or retrograde conduction observed during pacing or AVRT [8]. Patients with multiple accessory pathways were assigned to the anatomical subgroup according to the first successfully identified accessory pathway during the electrophysiological study. Figure 2 shows a case of manifest multiple APs (Figure 2A). During the AVRT, the atrial activation sequence exhibited two patterns (Figure 2D), and the earliest activation site varied from CS 5–6 to CS 1–2. Dual‐chamber mapping showed the earliest activation site on the posterior atrial septum (Figure 2B,D, Movie S2A), and RF ablation changed the tachycardia sequence. A second dual‐chamber mapping during ongoing tachycardia confirmed the new earliest activation site at the 3 o'clock position (Figure 2C, Movie S2B). Ablation at that site resulted in successful interruption of the accessory pathway at the 3 o'clock position.

### 2.5 Definition of Atypical Accessory Pathways

**FIGURE 3:**
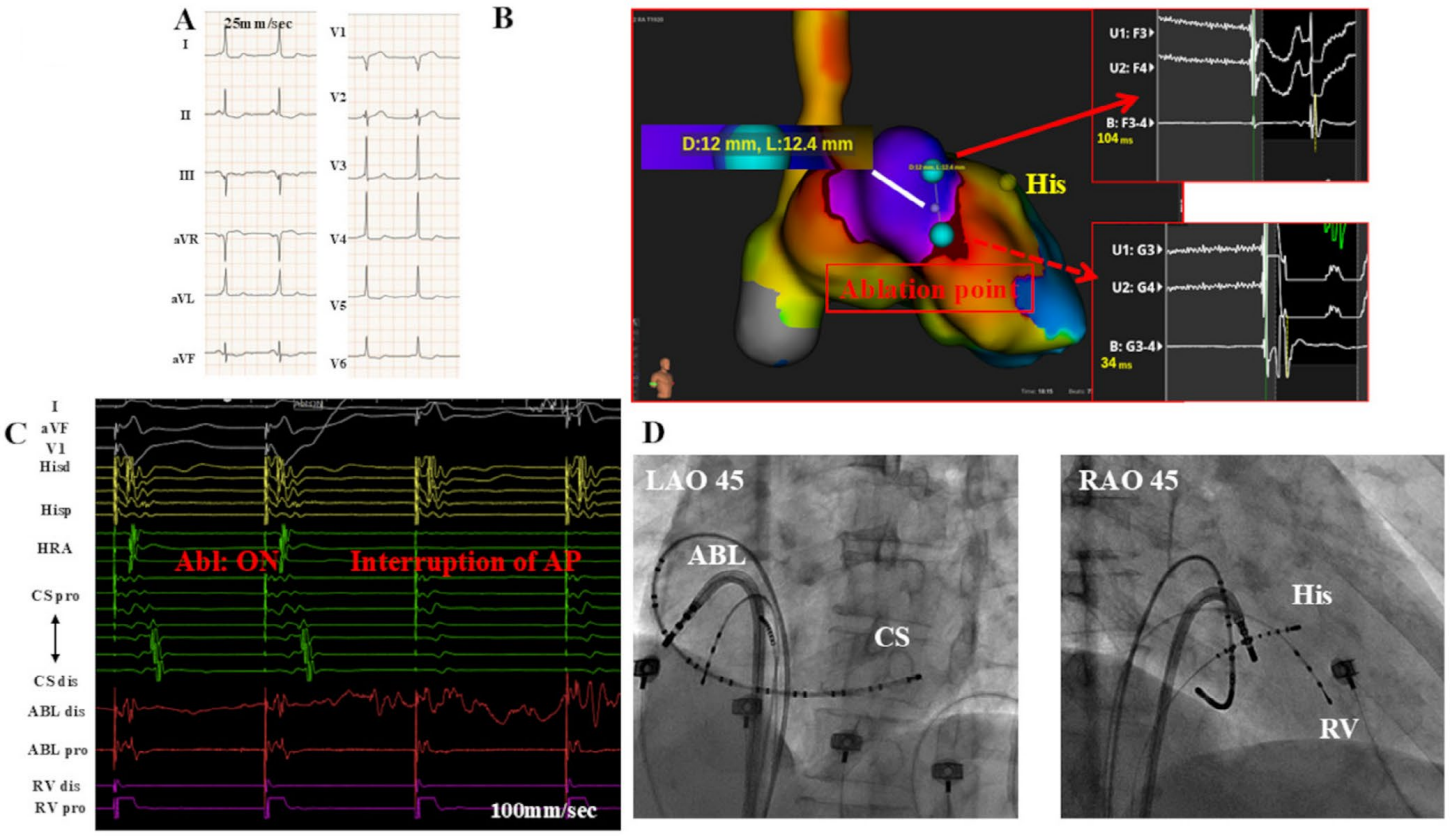
Representative case of an oblique accessory pathway. (A) The baseline 12‐lead ECG demonstrated manifest preexcitation, suggesting a septal AP. (B) The red arrow indicates the site of the earliest ventricular activation and its corresponding local electrogram during antegrade AP activation mapping. The red dotted arrow denotes the site of the earliest atrial activation during retrograde AP mapping as well as the ablation point. The linear distance between these two sites was 12 mm, and the curved distance measured on the 3D mapping surface was 12.4 mm. (C) IECGs recorded during ablation are shown. RF energy was delivered during RV pacing, resulting in successful interruption of the AP conduction within 1 s. (D) Fluoroscopic images demonstrate the catheter position at the site of successful ablation. Abbreviations are as in Figures 1 and 2.

Some patients exhibited atypical APs and those were defined by the following criteria:

An oblique pathway was defined in cases where the accessory pathway demonstrated bidirectional conduction (both antegrade and retrograde), and the anatomical distance between the earliest atrial and ventricular activation sites exceeded 10 mm on dual‐chamber mapping [4] constructed during AV and VA sequences, respectively [9, 10]. Figure 3 shows a representative case of a manifest oblique AP (Figure 3A). Dual‐chamber mapping was performed during both sinus rhythm and RV pacing. During sinus rhythm, the earliest atrial activation site was identified at the tricuspid annulus (TVA) in the 11 o'clock position, whereas during RV pacing, it shifted to the TVA in the 8 o'clock position (Figure 3B,C). To minimize the risk of AVB, RF ablation was delivered at the TVA 8 o'clock position, away from the His bundle recording site, successfully eliminating both antegrade and retrograde conduction through the AP (Figure 3B,C). The linear distance between the two earliest activation sites obtained from mapping was 12 mm, and the corresponding surface distance on the 3D mapping system was 12.4 mm. Based on these findings (Figure 3B), the AP was diagnosed as an oblique pathway.

### 2.6 Complex Ablation Approach for AP Elimination

Some patients exhibited recurrence of AP conduction that required elimination using a complex ablation approach. A complex ablation approach was defined as cases requiring applications from both atrial and ventricular aspects (e.g., supra‐ and sub‐valvular approaches) or from within the CS.

**FIGURE 4:**
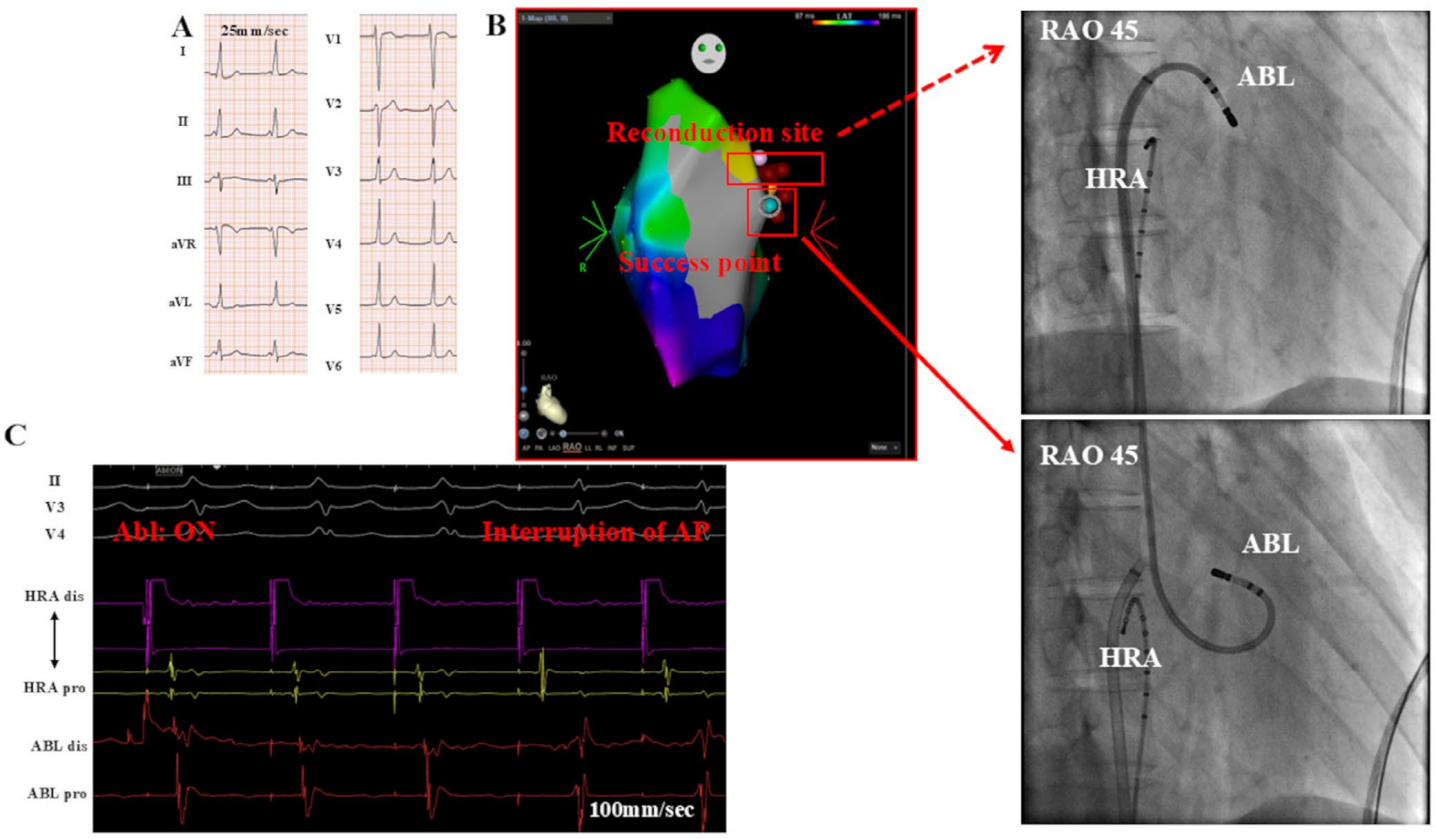
Representative case requiring a complex ablation approach. (A) The baseline 12‐lead ECG demonstrated manifest preexcitation, suggesting a right AP. (B) Ablation tags are displayed on the 3D mapping system. The red dotted arrows indicate the ablation tags created via the transfemoral approach, during which AP reconduction was observed during the procedure. In contrast, the red arrow shows the ablation tag delivered from the subvalvular aspect via the transjugular approach, which successfully achieved complete interruption of AP conduction. (C) IECGs recorded during successful ablation are shown. Radiofrequency energy was delivered during high right atrium pacing, resulting in successful interruption of AP conduction within 1.5 s. Abbreviations are as in Figures 1 and 2.

Figure 4 shows a case of a manifest AP (Figure 4A). The AP was located on the TVA in the 12 o'clock position. Although multiple RF applications were delivered via the transfemoral approach, the pathway repeatedly reconducted. Therefore, the approach was switched to the transjugular route, and RF energy was applied from the subvalvular aspect. The AP was immediately interrupted and did not reconduct thereafter (Figure 4B,C).

### 2.7 Statistical Analysis

The statistical analyses were performed using JMP Pro version 16.0 software (SAS Institute, Cary, NC, USA). Data are expressed as mean ± SD for parametric data, and continuous variables were compared using a *t*‐test for the parametric data and Mann–Whitney test for the non‐parametric data. The categorical data were compared by a chi‐square test. A receiver operating characteristic (ROC) curve analysis was utilized to evaluate the cutoff value for the optimal waiting period. Two‐sided *p* < 0.05 were considered statistically significant.

## 3 Results

A total of 128 cases (reconduction group [*n* = 23] vs. non‐reconduction group [*n* = 105]) were analyzed. A total of 130 accessory pathways were identified in 128 patients because two patients had multiple accessory pathways. Thirteen patients (10.2%) underwent repeat procedures (12 s procedures and 1 third procedure). The proportion of repeat procedures did not differ significantly between the reconduction and non‐reconduction groups (2/23 [8.7%] vs. 11/105 [10.5%], *p* = 0.80). Six patients (4.7%) were lost to follow‐up. Among the remaining 122 patients, the median follow‐up duration was 136 days (interquartile range [IQR], 56–605 days). Late recurrence was observed in 11 patients, including 5 of 23 patients (21.7%) in the reconduction group and 6 of 105 patients (5.7%) in the non‐reconduction group. The median time from the index ablation procedure to documented recurrence was 86 days (IQR, 42–291 days).

Table 1 shows the background characteristics, arrhythmia details, procedural details, and 3D mapping system.

**TABLE 1:** Patient characteristics.

	Reconduction (*n* = 23)	Non‐reconduction (*n* = 105)	*p*
Background characteristics			
Age (year)	46 ± 4	49 ± 2	0.41
Gender, Male, *n* (%)	17 (73.9%)	76 (72.4%)	0.88
Height (cm)	164.6 ± 1.9	165.9 ± 0.9	0.52
BMI	21.8 ± 0.7	22.4 ± 0.3	0.42
Coronary artery disease, *n* (%)	1 (4.4%)	3 (2.9%)	0.71
Hypertension, *n* (%)	4 (17.4%)	26 (24.8%)	0.45
Diabetes, *n* (%)	1 (4.4%)	6 (5.7%)	0.79
Hyperlipidemia, *n* (%)	3 (13.0%)	16 (15.2%)	0.79
History of heart failure, *n* (%)	0 (0%)	5 (4.8%)	0.99
Chronic kidney disease, *n* (%)	1 (4.4%)	4 (3.9%)	0.91
History of Af, *n* (%)	3 (13.0%)	16 (15.2%)	0.79
Arrhythmia details			
Accessory pathway location			
Left AP, *n* (%)	16 (69.6%)	71 (67.6%)	
Right AP, *n* (%)	3 (13.0%)	14 (13.3%)	
Septal AP, *n* (%)	4 (17.4%)	20 (19.1%)	
			0.98
Multiple/broad/oblique pathway, *n* (%)	7 (30.4%)	7 (6.7%)	0.0009
Manifest, *n* (%)	7 (30.4%)	42 (40.0%)	
Intermittent, *n* (%)	5 (21.7%)	13 (12.4%)	
Concealed, *n* (%)	11 (47.8%)	50 (47.6%)	
			0.44
Procedural details			
Complex ablation approach, *n* (%)	6 (26.1%)	7 (6.7%)	0.005
Total number of applications	7.2 ± 10.4	5.7 ± 5.4	0.30
Time to pathway interruption (sec)	8.6 ± 1.6	3.3 ± 0.7	0.004
Interval to the onset of CEA (msec)	21.6 ± 2.7	27.1 ± 1.2	0.97
Late recurrence, *n* (%)	5 (21.7%)	6 (5.7%)	0.013
Time to reconduction of AP (min)	16.7 ± 7.0		
Complication, *n* (%)	0 (0%)	3 (2.9%)	0.41
3D mapping system			
CARTO, *n* (%)	4 (17.4%)	42 (40.0%)	0.041
RHYTHMIA, *n* (%)	17 (73.9%)	60 (57.1%)	0.14
Ensite, *n* (%)	2 (8.7%)	3 (2.9%)	0.19

*Note:* The continuous variables are shown as mean ± SD and categorical variables as number (%).

Abbreviations: Af, atrial fibrillation; AP, accessory pathway; BMI, body mass index; CEA, continuous electrical activity.

### 3.1 Background Characteristics

There were no statistically significant differences in the baseline clinical characteristics between the reconduction group (*n* = 23) and non‐reconduction group (*n* = 105). The mean age was 46 ± 4 years in the reconduction group and 49 ± 2 years in the non‐reconduction group (*p* = 0.41). The proportion of male patients was comparable between groups (73.9% vs. 72.4%, *p* = 0.88). Other clinical parameters, including body mass index (BMI), and prevalence of coronary artery disease, hypertension, diabetes, hyperlipidemia, history of atrial fibrillation, heart failure, and chronic kidney disease, were also similar between groups.

### 3.2 Arrhythmia Details and Procedural Details

Radiofrequency ablation was used in 126 patients, whereas cryoablation was performed in two patients (one right anterolateral AP and one right anteroseptal AP) at the operator's discretion. Regarding arrhythmia characteristics, the distribution of AP locations (left AP, right AP, and septal AP) did not differ between the reconduction and non‐reconduction groups. The proportion of manifest, intermittent, and concealed pathways was also comparable between the two groups. On the other hand, the incidence of multiple, broad, or oblique accessory pathways was significantly higher in the reconduction group than in the non‐reconduction group (7[30.4%] vs. 7[6.7%], *p* = 0.0009).

**FIGURE 5:**
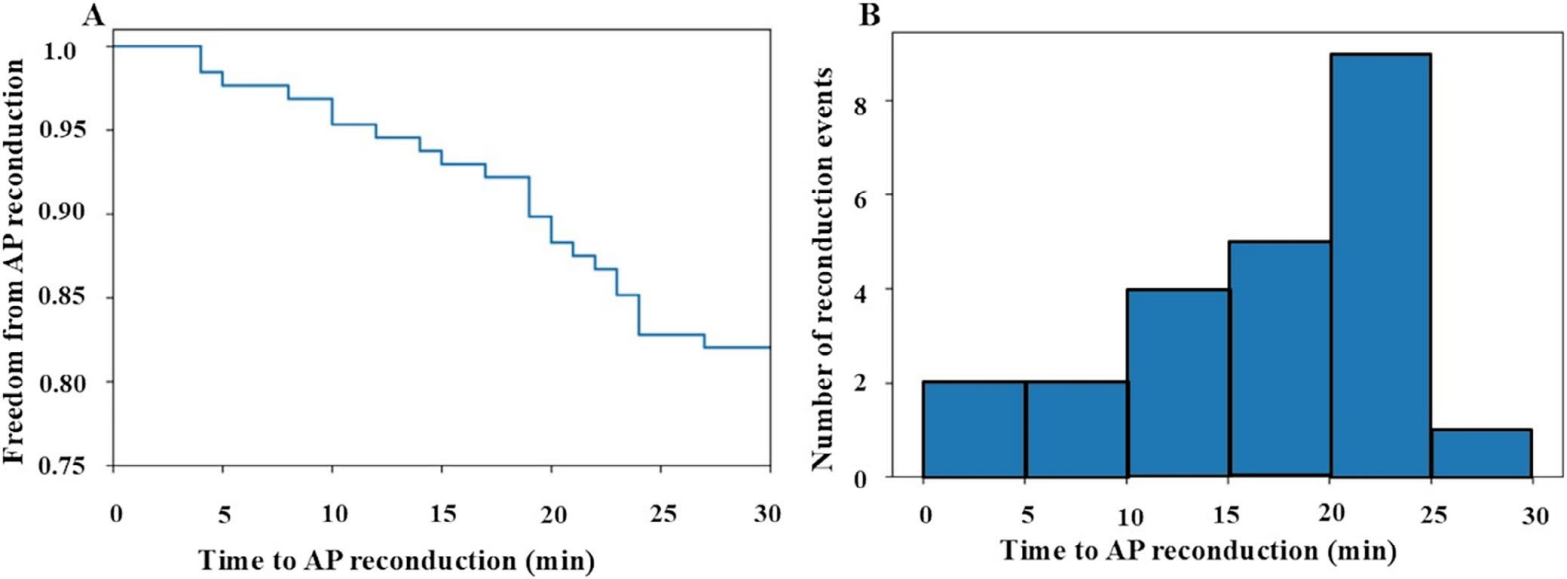
Temporal distribution of accessory pathway (AP) reconduction during the 30 min waiting period after apparent elimination of AP conduction. (A) Kaplan–Meier–style curve illustrating freedom from AP reconduction. Time was measured from the apparent elimination of AP conduction to the first detection of reconduction during the waiting period. (B) Histogram showing the distribution of the time to AP reconduction. Reconduction events occurred throughout the 30 min waiting period, with the highest frequency between 20 and 24 min.

With respect to procedural findings, patients in the reconduction group were more likely to require a complex ablation approach compared with the non‐reconduction group (6[26.1%] vs. 7[6.7%], *p* = 0.005). The total number of applications did not differ significantly between the reconduction and non‐reconduction groups (7.2 ± 10.4 vs. 5.7 ± 5.4, *p* = 0.30), whereas the time to pathway interruption was significantly longer in the reconduction group (8.6 ± 1.6 vs. 3.3 ± 0.7 s, *p* = 0.004). ROC curve analysis revealed an AUC of 0.60 for time to pathway interruption, with a cut‐off threshold of 5.57 s for predicting reconduction. In contrast, the electrogram advance recorded by the ablation catheter did not differ significantly between the groups (21.6 ± 2.7 vs. 27.1 ± 1.2 ms, *p* = 0.97). The mean time to reconduction of the APs was 16.7 ± 7.0 min, and the longest observed time to reconduction was 27 min. Among the 23 patients with intra‐procedural reconduction, 4 (17.4%) exhibited antegrade‐only reconduction, 18 (78.3%) exhibited retrograde‐only reconduction, and 1 (4.3%) exhibited both antegrade and retrograde reconduction. The Kaplan–Meier–style analysis demonstrated that AP reconduction occurred throughout the 30 min waiting period, with 15 of the 23 reconduction events (65.2%) detected 15 min or later after apparent elimination of AP conduction (Figure 5A). Histogram analysis showed that the highest frequency of reconduction occurred between 20 and 24 min (Figure 5B).

### 3.3 Subgroup Analyses Based on Accessory Pathway Type

Table 2A shows further investigating factors associated with intra‐procedural reconduction.

**TABLE 2A:** Comparative analysis within left accessory pathways.

	Reconduction (*n* = 16)	Non‐reconduction (*n* = 71)	*p*
Location of the accessory pathway			
Anterolateral, *n* (%)	3 (18.8%)	16 (22.5%)	
Lateral, *n* (%)	5 (31.3%)	33 (46.5%)	
Posterolateral, *n* (%)	6 (33.3%)	20 (29.2%)	
			0.43
Multiple/broad/oblique pathway, *n* (%)	5 (31.3%)	3 (4.2%)	0.0007
Procedural details			
Complex ablation approach, *n* (%)	3 (18.8%)	3 (4.2%)	0.038
Total number of applications	8.2 ± 12.4	5.0 ± 4.6	0.09
Time to pathway interruption (sec)	6.3 ± 0.9	3.3 ± 0.4	0.004
Interval to the onset of CEA (msec)	22.1 ± 3.3	25.9 ± 1.5	0.30
Late recurrence, *n* (%)	3 (18.8%)	2 (2.8%)	0.013

#### 3.3.1 Left Accessory Pathways (Table 2A)

Among patients with left AP, the distribution of AP locations did not differ significantly between the reconduction and non‐reconduction groups. However, multiple, broad, or oblique pathways were more frequently observed in the reconduction group (5[31.3%] vs. 3[4.2%], *p* = 0.0007). The reconduction group more often required a complex ablation approach (3[18.8%] vs. 3[4.2%], *p* = 0.038) and demonstrated a longer time to pathway interruption (6.3 ± 0.9 vs. 3.3 ± 0.4 s, *p* = 0.004). Late recurrence was also more frequent in the reconduction group (18.8% vs. 2.8%, *p* = 0.013).

#### 3.3.2 Right Accessory Pathways (Table 2B)

**TABLE 2B:** Comparative analysis within right accessory pathways.

	Reconduction (*n* = 3)	Non‐reconduction (*n* = 14)	*p*
Location of the accessory pathway			
Anterolateral, *n* (%)	3 (100%)	5 (35.7%)	
Lateral, *n* (%)	0 (0%)	1 (7.1%)	
Posterolateral, *n* (%)	0 (0%)	8 (57.1%)	
			N/A
Multiple/broad/oblique pathway, *n* (%)	0 (0%)	4 (28.6%)	0.29
Procedural Details			
Complex ablation approach, *n* (%)	2 (66.7%)	0 (0%)	0.001
Total number of applications	6.0 ± 3.6	4.9 ± 3.1	0.61
Time to pathway interruption (sec)	14.0 ± 5.1	1.7 ± 2.8	0.06
Interval to the onset of CEA (msec)	19.3 ± 6.0	29.0 ± 2.8	0.16
Late recurrence, *n* (%)	2 (66.7%)	1 (7.1%)	0.014

In patients with right AP, reconduction was observed exclusively in those with anterolateral accessory pathways (3[100%] vs. 5[35.7%]). The reconduction group more often required a complex ablation approach (2[66.7%] vs. 0[0%], *p* = 0.001) and demonstrated a longer time to pathway interruption (14.0 ± 5.1 vs. 1.7 ± 2.8 s), however, this difference did not reach statistical significance (*p* = 0.06). Late recurrence occurred more frequently in the reconduction group (66.7% vs. 7.1%, *p* = 0.014).

#### 3.3.3 Septal Accessory Pathways (Table 2C)

**TABLE 2C:** Comparative analysis within septal accessory pathways.

	Reconduction (*n* = 4)	Non‐reconduction (*n* = 20)	*p*
Location of the accessory pathway			
Anteroseptal, *n* (%)	0 (0%)	4 (20.0%)	
Mid‐septal, *n* (%)	0 0 (%)	0 (0%)	
Posteroseptal, *n* (%)	2 (100%)	16 (80.0%)	
			N/A
Multiple/broad/oblique pathway, *n* (%)	2 (50%)	0 (0%)	0.001
Procedural Details			
Complex ablation approach, *n* (%)	1 (25%)	4 (20%)	0.82
Total number of applications	4.3 ± 1.7	8.5 ± 7.9	0.31
Time to pathway interruption (sec)	20.8 ± 7.7	5.8 ± 4.5	0.11
Interval to the onset of CEA (msec)	20.8 ± 6.8	30.2 ± 3.3	0.23
Late recurrence, *n* (%)	0 (0%)	3 (15.0%)	0.41

*Note:* The continuous variables are shown as mean ± SD and categorical variables as number (%).

Abbreviations: RF, radiofrequency; WPW, Wolff–Parkinson–White.

For septal AP, reconduction was not associated with a particular pathway location. However, the reconduction group more frequently exhibited multiple, broad, or oblique pathways (2[50%] vs. 0[0%], *p* = 0.001). No significant differences were observed between the groups with respect to the need for a complex ablation approach, time to pathway interruption, or electrogram advance of the ablation catheter. Late recurrence was not observed in the reconduction group but occurred in 15.0% of the non‐reconduction group (*p* = 0.41).

### 3.4 Subgroup Analysis Within the Reconduction Group

Table 3 shows the subgroup analysis of arrhythmia and procedural details between patients with and without late recurrence within the reconduction group. Within the reconduction group, a total of five patients (22%) experienced late recurrence. The proportions of manifest, intermittent, and concealed pathways, as well as the prevalence of multiple, broad, or oblique pathways, were comparable between the two groups.

**TABLE 3:** Clinical characteristics of late recurrence vs. non‐recurrence in the reconduction group.

Reconduction group (*n* = 23)	late recurrence (*n* = 5)	Non‐late recurrence (*n* = 18)	*p*
Arrhythmia details			
Left AP, *n* (%)	3 (60%)	13 (72.2%)	
Right AP, *n* (%)	2 (40%)	1 (5.6%)	
Septal AP, *n* (%)	0	4 (22.2%)	
			N/A
Manifest, *n* (%)	2 (40%)	5 (27.8)	
Intermittent, *n* (%)	0	5 (27.8%)	
Concealed, *n* (%)	3 (60%)	8 (44.4%)	
			N/A
Multiple/broad/oblique pathway, *n* (%)	1 (20%)	6 (33.3%)	0.57
Procedural Details			
Complex ablation approach, *n* (%)	2 (40%)	4 (22.2%)	0.90
Total number of applications	12.6 ± 11.0	5.7 ± 10.1	0.20
Time to pathway interruption (sec)	11.0 ± 7.1	9.9 ± 4.0	0.06
Interval to the onset of CEA (msec)	21.0 ± 7.3	21.8 ± 3.9	0.93
Time to reconduction of AP (min)	17.2 ± 3.6	14.2 ± 1.9	0.47

*Note:* The continuous variables are shown as mean ± SD and categorical variables as number (%).

Abbreviation: AP, accessory pathway.

With respect to procedural details, the late recurrence group more frequently required a complex ablation approach (2[40%] vs. 4[22.2%], *p* = 0.90), a difference that was not statistically significant. The reconduction group with late recurrence demonstrated a higher mean total number of applications compared with those without recurrence (12.6 ± 11.0 vs. 5.7 ± 10.1, *p* = 0.20), which also did not reach statistical significance. Similarly, the time to pathway interruption was longer in patients with late recurrence (11.0 ± 7.1 vs. 9.9 ± 4.0 s, *p* = 0.06), without statistical significance. Electrogram advance at the ablation catheter was comparable between the two groups (21.0 ± 7.3 vs. 21.8 ± 3.9 ms, *p* = 0.93). The time to accessory pathway reconduction did not differ significantly (17.2 ± 3.6 vs. 14.2 ± 1.9 min, *p* = 0.47).

## 4 Discussion

### 4.1 Major Findings

The major findings are summarized as follows:

(1) Intra‐procedural reconduction was observed in 18% of patients (23/128) and was associated with a higher observed rate of late recurrence (21.7% vs. 5.7%, *p* = 0.013). The mean time to AP reconduction was approximately 16.7 ± 7.0 min. (2) In left APs, reconduction was strongly associated with the presence of multiple, broad, or oblique pathways and longer time to pathway interruption. (3) In right APs, reconduction occurred exclusively in anterolateral APs, which also required more complex ablation approaches. Notably, late recurrence was observed in two‐thirds of these cases.

### 4.2 Considerations Regarding Accessory Pathway Location and Ablation Characteristics

Previous studies have suggested that a higher number of applications, prolonged procedural duration, and complex ablation characteristics are associated with increased rates of late recurrence [11, 12, 13]. In particular, right‐sided, epicardial, or multiple accessory pathways have been shown to be associated with significantly lower procedural success rates and inferior long‐term outcomes [1, 14, 15]. Previous studies have demonstrated that a shorter time to accessory pathway interruption during catheter ablation is significantly associated with a reduced risk of late recurrence [16, 17]. In our study, these characteristics were also correlated with intra‐procedural reconduction, indicating that such anatomical and procedural features may not only impact chronic outcomes but also influence intra‐procedural reconduction.

Furthermore, among patients with septal APs, the time to pathway interruption did not differ significantly between the reconduction and non‐reconduction groups. Although operators may adopt a more cautious ablation strategy for septal APs because of their proximity to the normal conduction system and the associated risk of AVB [18, 19], the small number of reconduction events in this subgroup precludes definitive conclusions regarding this association.

### 4.3 Clinical Implications

(1) Reconduction is a measurable procedural phenomenon that significantly correlates with late recurrence and should not be overlooked as a transient. (2) A longer time to initial AP interruption, multiple or anatomically complex pathways may identify patients at increased risk of intra‐procedural reconduction. These parameters may serve as procedural red flags, prompting more cautious post‐ablation observation. Recognition of these characteristics may help identify patients who require closer intraprocedural observation and facilitate detection of AP reconduction. The additional time‐distribution analysis demonstrated that the mean time to AP reconduction was 16.7 ± 7.0 min. Furthermore, 65.2% of reconduction events were detected 15 min or later, with the highest frequency occurring between 20 and 24 min. These findings indicate that AP reconduction is not confined to the early waiting period but may occur throughout the 30 min observation period. Furthermore, in cases presenting multiple risk factors for intra‐procedural reconduction—such as longer time to interruption, complex or oblique pathways—an extended waiting period approaching 30 min may be considered to ensure durable elimination of the AP.

### 4.4 Study Limitations

Several limitations of this study should be acknowledged. First, this was a single‐center retrospective study, which may have introduced selection bias and limited the generalizability of our findings. Second, the relatively small number of reconduction events, particularly in the right‐sided and septal AP subgroups, limited the statistical power of these subgroup analyses. Third, repeat procedures were included, and although their frequency was similar between the reconduction and non‐reconduction groups, they may have introduced some procedural heterogeneity. Fourth, the recorded time to AP reconduction reflected the time of detection rather than the exact time of recovery because AP conduction was assessed intermittently during the waiting period. Finally, follow‐up duration varied among patients, and the limited number of late recurrence events means that the observed association between intra‐procedural reconduction and late recurrence should be considered exploratory.

## 5 Conclusion

Intra‐procedural reconduction of previously interrupted APs is a clinically relevant phenomenon associated with a significantly increased risk of late recurrence following catheter ablation of accessory pathways. Reconduction was more likely to occur in cases with multiple, broad, or oblique pathways, a longer time to pathway interruption, and those requiring complex ablation strategies. Recognition of these characteristics may help electrophysiologists identify patients in whom a prolonged waiting period or additional ablation during the index procedure may be considered.

## Author Contributions

Wataru Sasaki, Hitoshi Mori, and Ritsushi Kato provided the study concept and design; Wataru Sasaki and Hitoshi Mori drafted the manuscript; Wataru Sasaki, Kei Matsumoto, Naomichi Tanaka, Daisuke Kawano, Tsukasa Naganuma, Masataka Narita, and Kazuhisa Matsumoto contributed to the data analysis and interpretation. Yoshifumi Ikeda, Takahide Arai, Shintaro Nakano, and Ritsushi Kato reviewed and revised the manuscript draft.

## Funding

The authors have nothing to report.

## Ethics Statement

The study protocol was approved by the hospital's institutional review board (IRB number; 2025–061).

## Conflicts of Interest

The authors declare no conflicts of interest.

## Supporting information


**Movie S1:** Dual‐chamber mapping of a broad accessory pathway.

**Movie S2A:** Initial dual‐chamber mapping demonstrating the first accessory pathway.

**Movie S2B:** Repeat dual‐chamber mapping demonstrating the second accessory pathway.

**Figure S1:** Definition of the time to interruption (A) For antegrade AP ablation, time to interruption was measured from the onset of radiofrequency delivery to the QRS complex where the Δ wave disappeared on the surface 12‐lead ECG. (B) For retrograde AP ablation, time to interruption was defined as the interval from the onset of radiofrequency delivery to the atrial electrogram immediately following AP interruption. ABL, ablation; AP, accessory pathway; ECG, electrocardiogram.

**Figure S2:** Definition of the interval to the onset of CEA (A) Manifest AP during sinus rhythm showing the interval from delta‐wave onset to CEA. (B) Retrograde accessory pathway conduction during ventricular pacing showing the interval from the earliest annular atrial activation to CEA. CEA, continuous electrical activity.

## Data Availability

The data that support the findings of this study are available on request from the corresponding author. The data are not publicly available due to privacy or ethical restrictions.
